# Long-term characteristics of soil respiration in a Korean cool-temperate deciduous forest in a monsoon climate

**DOI:** 10.1080/19768354.2018.1433234

**Published:** 2018-01-30

**Authors:** Ji Young Eom, Seok Hee Jeong, Jung Hwa Chun, Jae Ho Lee, Jae Seok Lee

**Affiliations:** a Department of Biological Science, Konkuk University, Seoul, Korea; b Division of Forest Ecology, National Institute of Forest Science (NIFoS), Seoul, Korea; c Division of Ecosystem Services & Research Planning, National Institute of Ecology, Seocheon, Korea

**Keywords:** Soil respiration, long-term measurement, AOCC system, cool-temperate deciduous forest, monsoon climate

## Abstract

Analysis of relationship between soil respiration and environmental factors has become essential for understanding changes in ecosystem carbon cycles under global warming. However, rough predictions have been made that soil respiration will increase with increasing temperature, but long-term data to support this theory were scarce. We measured soil respiration and environmental factors continuously using an automatic open-closed chamber system in a Korean cool-temperate forest from 2004 to 2016 to ascertain the reliability of this prediction and to more accurately predict changes in carbon cycle. Average air and soil temperatures were 11.0°C and 10.2°C. The increase in temperature was greater in winter (the inactive period for soil respiration) than in summer (the active period). Additionally, precipitation decreased sharply because of patter changes in 2012, and through 2016, it was approximately 69% of the previous period. Effect of precipitation on soil respiration was expected to be larger than temperature because the change in precipitation appeared in summer. Soil respiration exhibited a significant decline in 2012 because of precipitation. From 2004 to 2011, it averaged 344.4 mgCO_2 _m^−2 ^h^−1^ and from 2012 to 2016 the average was 205.3 mgCO_2 _m^−2 ^h^−1^. This phenomenon hasn’t been detected in short-term studies, suggesting that the prediction of previous studies is inaccurate. Additionally, to predict future ecosystem carbon cycle changes in a cool-temperate monsoon climate, changes in precipitation pattern should be regarded as equally important to temperature, and the prediction cannot be based solely on temperature. Therefore, long-term and continuous measurements are needed with consideration of the effects of both precipitation and temperature.

**Abbreviations:** Rs: soil respiration; Ts: soil temperature; Ta: air temperature; AOCC: automatic open/closed chamber

## 1. Introduction

Enormous amounts of carbon, approximately twice that of the atmosphere and three times that of vegetation, is stored in the soil of forest ecosystems (Schimel et al. 1996). Soil organic carbon is increased through input of litter from vegetation and decreased by the emission of CO_2_ from the soil, which is defined as soil respiration (Rs). In general, Rs increases exponentially with rising temperature (Wang et al. 2006; Wu et al. 2006). Therefore, understanding the carbon dynamics of soil under global warming scenarios has been recognized as one of the most crucial components for assessing the ability of ecosystems, as a part of the global carbon budget, through Rs (Bernhardt et al. 2006). For these reasons, data are being collected to understand the characteristics of soil carbon cycles in forest ecosystems in various climates and to predict changes in soil carbon cycles following environmental changes.

To predict changes in the global carbon cycle, it is necessary to analyze the relationship between the characteristics and environmental factors for various ecosystems (Yuste et al. 2003). Temperature and moisture conditions are currently considered to be the important factors in the soil carbon cycle because of their influence on Rs (Raich et al. 2002); however, correlations between Rs and these factors have been difficult to define in tropical and polar climate regions. In the case of the tropical regions, effects related to changes in moisture conditions because of high temperature can be identified, but the reaction at low temperature cannot be observed. Conversely, in the polar regions, the response to high temperature is difficult to ascertain because low-temperature conditions are predominant. In this regard, the region of cool-temperate monsoon climate in Asia has a wide range of temperature and moisture conditions ranging from low to high temperature and from dry to moist. Therefore, it has the advantage of being able to demonstrate the influence of temperature and moisture on important soil carbon cycle regulators.

The cool-temperate deciduous forests in Korea experience heavy rain accompanied by typhoons (Changma) between June and August because of the monsoon climate (Chae 2011). During this period, environmental changes occur, such as rapid changes in soil moisture content and temperature because of decreases in solar radiation, which also changes Rs (Kwon et al. 2010). In addition, many researchers predicted that monsoon climate regions will experience a greater frequency and magnitude of precipitation during the Changma, with temperatures rising under global warming scenarios (Yun et al. 2008) and eventually a transitory, but significant, response in the carbon balance of the ecosystem will occur (Lee et al. 2004). Considering the changes in other mutually related environmental factors with changes in future precipitation, it has become essential to determine regional and global responses of carbon balance to the changing monsoon climate in Asia (Kwon et al. 2010).

To address this issue, research has been conducted in Korea, affected by the monsoon climate. Several studies conducted to date have focused on the growing season during which Rs is most active because of the appropriate environmental conditions for the growth of organisms (Rochette and Flanagan 1997; Kelting et al. 1998; Shi et al. 2011). Most of the other studies periodically measured Rs for periods of less than 2 years (longer than a growing season), but did not take measurements during winter when it is difficult to measure Rs because of weather conditions. Rs, which is not directly measured, is estimated using the indirect method, assuming that Rs in November is the same as that in winter or by using a regression equation between the soil temperature (Ts) and Rs (Kang et al. 2003; Yuste et al. 2003; Noh et al. 2010). In many studies, most Rs measurements were conducted using the portable chamber at a specific time (11:00–15:00), once or twice a month (Lin et al. 1999; Noh et al. 2010). The measurements can be affected by researchers because they are moved for measurements, and are affected by temporal and spatial conditions. In addition, this method does not reflect the average of data collected in various environments, such as after rainfall events or at nighttime, when Ts is lowered. This method can lead to distortion of the results because it yields averages based on limited temporary values (Davidson et al. 1998). Therefore, in short-term and discontinuous studies, researchers have not observed an immediate response of Rs to environmental changes, such as rainfall. Estimated annual soil carbon emissions based on these data does not reflect the environmental changes because of climate change in real time, and therefore, there are significant differences between the measured and actual values. For example, in a *Quercus mongolica* forest in Korea, Yu (2011) measured periodic but discontinuous Rs using the portable chamber method, and Joo et al. (2012) performed continuous measurements using an automated chamber system. Yu (2011) reported annual soil carbon emission of 6.8 tCha^−1 ^y^−1^, whereas Joo et al. (2012) reported it was 11.36 tCha^−1 ^y^−1^. The latter showed a significant difference of approximately 1.7 times that of the former. This indicates that the result of continuous measurements is different from the result of discontinuous measurements, because it reflects the immediate changes in Rs because of temporary environmental changes that cannot be detected by discontinuous measurements. Therefore, to understand and more precisely predict the carbon cycle, it is necessary to conduct long-term research using 24-hour real-time observations and a measurement method that can obtain continuous data for one year, instead of during growth period. However, because of the lack of technical expertise in automated measuring instruments needed to use this method, most studies have focused on rough predictions of Rs using short-term and discontinuous data collected over two to three years.

The goal of this study was to collect Rs data reflecting various environments in a Korean cool-temperate deciduous forest in a monsoon climate from 2004 to 2016 using a system, which is effective for continuous measurement. Using the data, we analyzed long-term characteristics of Rs and environmental factors a cool-temperate deciduous forest in the monsoon climate that have been difficult to determine with previous short-term observations.

## 2. Methods

### 2.1. Site description

The study site (37°45′25.37″N, 127°09′11.62″S, 340 m above mean sea level) is a natural broad-leaved deciduous forest located in Gwangneung, Gyeonggi-do, South Korea. The mean air temperatures (Ta) during the coldest and warmest months were –5.2°C and 24.7°C, respectively. According to Koppen’s climate classification, the climate of this study site was in the baseline of the cool and temperate climates (Yun et al. 2012). In addition, approximately 65% of annual precipitation was concentrated during summer by the monsoon climate. The dominant species were *Carpinus laxiflora*, *Q. serrata*, and *C. cordata*. According to the theory of climate climax in which the sere of forests obtains the climax forest, such as *C. laxiflora* and *C*. *cordata* through *Quercus* (Lee et al. 1992), the forest was a climax forest or primeval forest. In Korea, communities of *C. laxiflora* conserved on a large scale are unusual (Park et al. 2009). In addition, the forest has limited access to minimize environmental disturbance by humans. Thus, the forest could be characterized as a standard forest ecosystem in the natural state in the cool-temperate monsoon climate. Therefore, the forest was judged the most suitable site to measure the long-term changes in CO_2_ flux in the cool-temperate monsoon climate zone.

### 2.2. Soil respiration (Rs)

To measure Rs, we used an automatic open-closed chamber (AOCC) system (Suh et al. 2006). This system consisted of three large parts (chamber, pump, and timer). The pump sequentially sends air to the chambers by a signal from the timer, and the air in the chamber is returned to the infrared gas analyzer (IRGA; LI-820, Li-Cor, Lincoln, Nebraska, USA). The CO_2_ concentration measured in the IRGA is averaged every 30s and recorded by a data logger (CR10X, Campbell Scientific, Logan, Utah, USA) every 2 min. If no electrical events occur, the AOCC system stably repeats measurements throughout the day and provides accurate daily estimates of soil CO_2_ efflux despite temporal fluctuation (Jensen et al. 1996; Fang and Moncrieff, 1998). Therefore, it is an appropriate instrument for long-term surveys to continuously collect real-time data on Rs with minimal disturbance by researchers.

From 2004 to 2010, Rs was measured based on the open-flow method, which measures air inlet and outlet. For details, see Suh et al. (2006). Using the CO_2_ concentration data obtained by the AOCC system based on the open-flow method, Rs is calculated by using the following equation:

(1) Rs=(aLρ)/A

where *a* is the difference in CO_2_ concentrations between the ambient and chamber air measured at the air inlet and outlet of the chamber, *L* is the flow rate, *ρ* is the density of CO_2_, and *A* is the surface area in the chamber.

From 2011, the method was switched from the open-flow method to the closed dynamic method. In addition, there was a slight difference in the equation for calculation Rs by each method. Rs based on the closed dynamic AOCC was calculated using the following equation:

(2) Rs=(aVρ)/A

where *a* (▵CO_2_/Δ*t*) is the time rate of change of the CO_2_ concentration in the chamber, and *V* is the volume of the chamber, and *ρ* and *A* are the same as in the Equation (1).

On the forest floor, six chambers were installed with reference to tree positions. Small litter and twigs were left in the chambers and large objects were removed. All chambers were left at the study site during the entire study period.

### 2.3. Environmental factors

Because recent climate change is apparent in temperature and precipitation, and Rs on a global scale is highly correlated with annual temperature, annual precipitation (Raich and Schlesinger 1992), we measured Ta, Ts, and precipitation.

To measure Ta, a thermocouple (T-CC, 0.32 mm, Ninomiya) was installed at 1.2 m above the ground near the system box containing the IRGA. The thermocouple was installed in a white shell to block solar radiation, and Ts was measured near the chambers using the same as that used for the Ta measurement. Because the organic carbon content in soils used for Rs was the largest in the surface layer (Persson 1980), Ts was measured at a depth of 5 cm. To measure the precipitation, we installed a rain gauge (TE525MM, Texas Inc., USA) horizontal to the floor. All environmental factors were measured every 30 s, averaged over 2-minute intervals, and automatically logged by the data loggers used for recording Rs.

## 3. Results and discussion

### 3.1. Environmental factors

**Figure 1.**
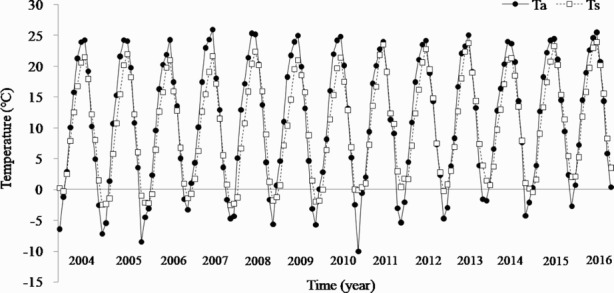
Long-term changes in monthly air (Ta) and soil (Ts, 5 cm depth) temperatures in a Korean cool-temperate deciduous forest from 2004 to 2016.

The average Ta was 11.0°C during the study period (Figure 1). In 2004, the annual Ta was 10.2°C, which was lower than the average Ta in the study period, and it decreased to 9.2°C in 2005, and then returned to the previous level in 2006. From that time until 2009, Ta was similar to the average for the overall study period. It decreased from 2010 to 2011 being 10.1°C. Conversely, beginning in 2012, the average Ta was higher than the overall average during the study period and gradually increased to a maximum Ta of 12.7°C in 2016. Thus, the annual Ta increased and the growth rate was approximately 0.21°C y^−1^ during the entire period. The highest Ta in summer increased at a rate of approximately 0.09°C y^−1^, whereas the lowest Ta in winter increased at a higher rate of approximately 0.29°C y^−1^. This result agreed with a study indicating that the summer Ta increase, which was highly affected by precipitation because of the monsoon climate, was small, whereas the Ta in Korea has been steadily increasing over the past 30 years (Heo and Kwon 2007). It is expected that the increase in annual Ta during the winter, which is the inactive period for Rs, wouldn’t have a significant effect on Rs. In addition, the Ta indicated general seasonal variation. The Ta of the coldest month (January) was –5.2°C and it increased steadily from April, and the Ta of the warmest month (August) was 24.7°C, after which it decreased again.

The average Ts during the study period was approximately 10.2°C, which increased by approximately 0.21°C y^−1^, similar to Ta (Figure 1). The annual Ts in 2004 was 10.1°C, which decreased to 8.6°C in 2005, and remained consistent until 2010. However, the Ts in 2011 increased rapidly to 10.8°C, which is 0.6°C higher than the average during the overall period, and then gradually increased to 12.6°C, the highest Ts in 2016. Similar to Ta, Ts showed an increase of 0.16°C y^−1^ in summer and exhibited an increase of 0.38°C y^−1^ during winter. Among the environmental factors, Ts had the greatest effect on Rs; however, the response of Rs to the increasing trend in Ts was likely not significant. In addition, Ts exhibited a typical seasonal pattern. For monthly Ts values, the minimum value (−0.7°C) occurred in January and it increased steadily from April, in August it was 22.3°C, and it steadily decreased from September to 1.9°C in December.

**Figure 2.**
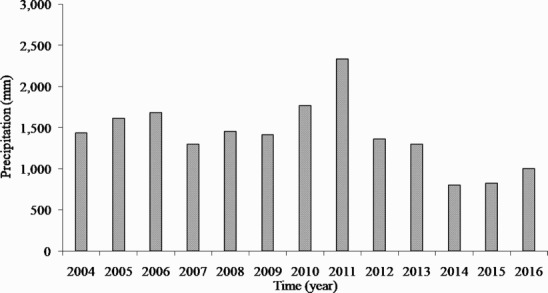
Variation of annual precipitation in a Korean cool-temperate deciduous forest from 2004 to 2016. Precipitations were shown a relatively low value for period from 2012 to 2016 compared with it from 2004 to 2011.

During the study period, there was a change in precipitation, which occurred at a similar time as temperature. However, precipitation fluctuated more widely than temperatures (Figure 2). By 2010, the average of annual precipitation was 1526 mm, which was higher than the average precipitation of 1408 mm during the study period. However, 2011 was an unusual year, with annual precipitation of 2337 mm. After 2011, precipitation decreased sharply to 1362 mm in 2012, decreased again to 806 mm in 2014, and then gradually increased until 2016. Thus, the average precipitation was 1059 mm from 2012 to 2016, corresponding to approximately 69% of the average precipitation from 2004 to 2010. This rapid change was a phenomenon not detected in previous studies, and we compared the measured rainfall with that at the Dongducheon observatory. The average of annual precipitation was 1506 mm from 2004 to 2016 according to the Dongducheon observatory. Subsequently, annual precipitation increased rapidly to 2311 mm in 2011 and decreased to 1384 mm again in 2012. In 2014, it decreased to 742 mm and then gradually increased. The results of this study were deemed reliable because they were similar to that of the Dongducheon observatory of the Korea Meteorological Administration. In addition, from 2012, the pattern of rainfall changed and included a dry period, except for short-term heavy rainfall. Before of the change in the pattern from 2004 to 2010, precipitation exceeding 100 mm was recorded evenly from May to September. However, from 2012 when the pattern changed, it was concentrated in short term events from July to August. Although precipitation was concentrated after 2012, it was only 68% of the monthly precipitation from July to August from 2004 to 2010. Furthermore, in 2011, there was record precipitation, with a total of 1986 mm observed during the summer (June to August). This was approximately 2.5 times the annual precipitation of 2014, the period of the least precipitation during the study period. In this study, the increase in temperatures appeared in winter but the fluctuation was very narrow compared to the change in the precipitation pattern. Precipitation exhibited a remarkable change in summer. Therefore, according to the results of previous studies (Chae 2011), it was expected that changes in precipitation and its pattern will cause changes in the hydrological cycle of forest soils, resulting in changes in Rs.

### 3.2. Characteristics of Rs

**Figure 3.**
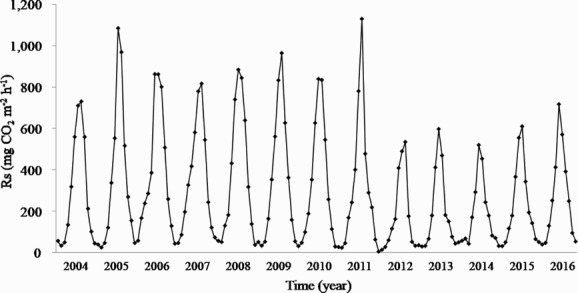
Long-term changes in monthly soil respiration (Rs) in a Korean cool-temperate deciduous forest from 2004 to 2016. Rs were shown a relatively low value period from 2012 to 2016 compared with it from 2004 to 2011.

Rs was measured continuously from 2004 to 2016 and averaged 290.0 mgCO_2 _m^−2 ^h^−1^ (Figure 3). The maximum of annual value was 383.8 mgCO_2 _m^−2 ^h^−1^ in 2006, and the minimum was 173.8 mgCO_2 _m^−2 ^h^−1^ in 2012. Rs was within the range of 127–592 mgCO_2 _m^−2 ^h^−1^for deciduous forests specified by previous research (Raich and Schlesinger 1992; Bond-Lamberty et al. 2004). Rs rate exhibited a remarkable decrease at the time when environmental factors changed, although it was more evident than environmental factors. This phenomenon wouldn’t be detected in the short-term studies. Based on these results, we analyzed period 1 from 2004 to 2011 before the changes in factors, and period 2 from 2012 to 2016 when factors changed. Period 1 had annual fluctuations in Rs, but annual Rs ranged from 293.2 to 383.8 mgCO_2 _m^−2 ^h^−1^, with a period average of 344.4 mgCO_2 _m^−2 ^h^−1^. However, in period 2, Rs abruptly decreased, and annual Rs range was 173.8–251.3 mgCO_2 _m^−2 ^h^−1^, and the period average was 205.3 mgCO_2 _m^−2 ^h^−1^. The average of period 1 was approximately 1.7 times that of the average of period 2. The average of period 1 was similar to the result of 330 mgCO_2 _m^−2 ^h^−1^ of Son et al. (1994) and 315 mgCO_2 _m^−2 ^h^−1^ of Heo (2015), whose study were conducted in deciduous forests near this study’s site. However, the difference between the two studies and the average of period 2 was approximately five times larger than the difference from period 1. Son et al. (1994) measured Rs every three weeks over only six months using the soda-lime technique, and Heo (2015) measured it monthly over 12-month using a portable CO_2_ sensor that has been used frequently in previous studies. They measured Rs temporarily, instead of measuring continuously 24-hour, and calculated daily or monthly Rs. In the case of such discontinuous measurements, it is possible to overestimate Rs of the natural state by measuring at the time of increasing temperature in the forest or physical disturbance by the researchers. In addition, because Rs was measured over a short-term period (less than 1-year), researchers couldn’t observe the rapid decrease in Rs and precipitation recorded in this study. Thus, although climate and vegetation were similar, the above two results are different from this study. In addition, the importance of long-term and continuous measurement in the present study to accurately elucidate the characteristics of Rs was recognized. Furthermore, the seasonal variation in Rs was high in summer and low in winter, closely paralleling Ts. The monthly Rs exhibited a minimum of 42.3 mgCO_2 _m^−2 ^h^−1^ in January when Ts dropped to –0.7°C. After which, Rs rose sharply from May, when Ts increased substantially, and the maximum of 745.5 mgCO_2 _m^−2 ^h^−1^ occurred in August when Ts was highest (22.3°C). Together with the decrease in Ts, it dropped again from September and decreased to an average of 51.3 mgCO_2 _m^−2 ^h^−1^ in December.

Based on the measured Rs, the annual soil carbon emission was 6.9 tCha^−1 ^y^−1^. Although there was slight variation, it was at a similar level in period 1 and emitted an average of 8.2 tCha^−1 ^y^−1^. However, in period 2, only an average of 4.9 tCha^−1 ^y^−1^ was emitted, which was approximately half of period 1. These changes were similar to changes in precipitation rather than temperatures. Period 1 exhibited similar trends with no significant changes in precipitation and soil carbon emissions. In period 2, however, soil carbon emissions decreased dramatically as precipitation decreased. This is consistent with a study in which a change in the precipitation caused a change in the soil water and eventually led to changes in soil carbon emission (Dairaku et al. 2004).

Many studies have been conducted on this study’s site because it is a representative forest in the middle of the Korean Peninsula with a cool-temperate monsoon climate. If the study was conducted only from 2006 to 2008, when soil carbon emission was high, the annual soil carbon emission would have been estimated to be 8.8 tCha^−1 ^y^−1^, which would have been the representative value for this region. However, if the study was conducted from 2012 to 2014, when the soil carbon emission was relatively low, the annual soil carbon emission would have been 4.4 tCha^−1 ^y^−1^. The soil carbon emission during period of high emission was twice that of the period of low emission. If the researchers conducted the study only during one of the two periods, they wouldn’t detect the change in soil carbon emission that occurred in conjunction with the environmental change, and would have misjudged the representative value of the area. It could have been used to assess the capacity of carbon sink in Korean forest ecosystems or to build a carbon cycle model as a basis, which would have resulted in erroneous results. In addition, discontinuous measurement of Rs reduces the accuracy and reliability of estimating annual soil carbon emission because it does not reflect changes in daily Rs and environmental changes, such as precipitation over the long term. However, by measuring Rs continuously for 365 days and 24 h a day, and estimating annual soil carbon emission based on this, the change of Rs related to the environmental changes, such as the daily weather conditions can be determined accurately.

**Figure 4.**
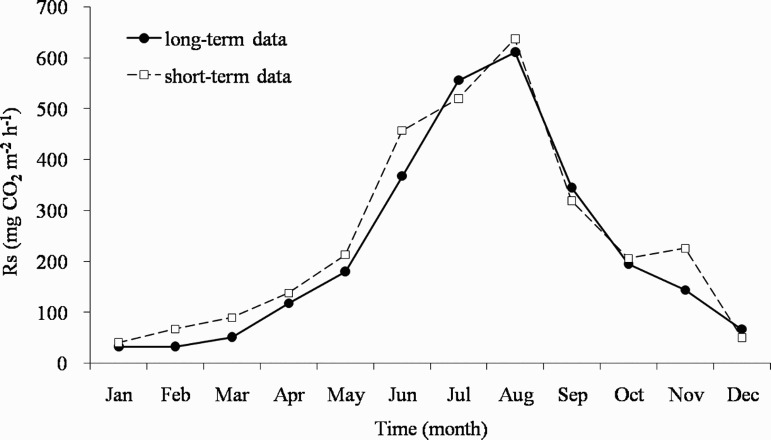
Comparison of soil respiration (Rs) from long-term data (total data average over 2015) and short-term data (at 14:00 on the 16th of each month in 2015) collected from a cool-temperate deciduous forest in Korea. The soil carbon emission calculated from the short-term data was higher than that of the long-term data, which indicated that the short-term data overestimated Rs relative to the long-term data.

To confirm this, we compared the long-term measurement data with the short-term measurement data using the data from 2015 (Figure 4). The continuous data for 2015 were used as the data for the long-term measurements and the data at 14:00 on the 16th of each month were used as the data for the short-term measurements. The daily Rs based on the short-term measurements was calculated by assuming that the measured hourly Rs was repeated for the entire day, similar to previous studies using short-term measurements. As a result, the daily Rs was calculated to be 246.5 mgCO_2 _m^−2 ^h^−1^, which was 22 mgCO_2 _m^−2 ^h^−1^ higher than the long-term measurement. Based on this, the annual soil carbon emission according to the short-term data was 5.9 mgCO_2 _m^−2 ^h^−1^ and it was 0.5 mgCO_2 _m^−2 ^h^−1^ higher than that calculated from the long-term data. This indicated that the short-term measurements overestimated Rs by approximately 10% relative to the long-term measurements. Therefore, continuous and long-term measurements are essential for the elucidation of soil carbon dynamics in the forest based on the cool-temperate monsoon climate, rather than discontinuous and short-term measurements.

### 3.3. Relationship between Rs and environmental factors

**Figure 5.**
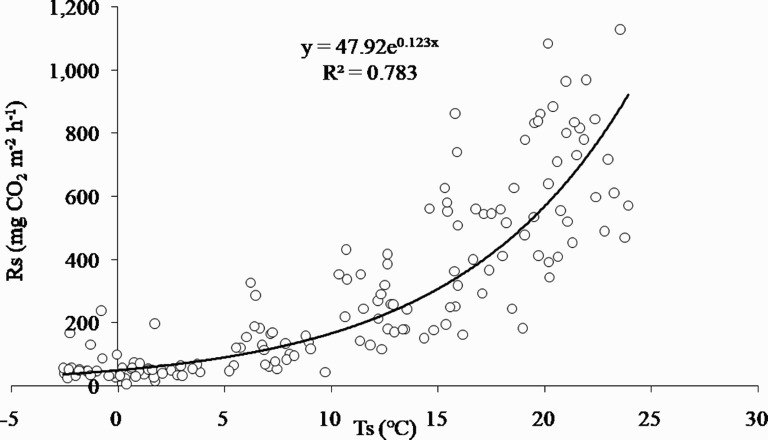
Correlation between monthly soil respiration (Rs) with monthly soil temperature (Ts) from 2004 to 2016 in a Korean cool-temperate deciduous forest. It showed a relatively high and positive correlation, but there were differences between Rs values at similar Ts.

A variability of Rs has been reported to be caused by changes in Ts, and it is known that Rs exponentially increases with increasing Ts in a sufficient soil moisture (Hubbard et al. 2005). Agren et al. (1991) reported that when Ts is in the range of 10 to 30°C, the microbial activity and Rs increases, whereas Rs decreased in the other Ts range. Furthermore, in this study, approximately 85.6% of the annual soil carbon emission occurred between May and October when Ts was in the range of 10 to 30°C. The monthly data were analyzed using a regression analysis and the exponential function (Davidson et al. 1998), which revealed a relatively high and positive correlation (Figure 5). The sensitivity of Rs to Ts, defined as the Q_10_, was calculated to be 3.5. It was similar to 3.8 (Heo 2015) but differed from 5.2 (Mo 2003), which were the results from deciduous forests near our study site. The average Ts in Mo (2003) was 9.8°C, which was lower than that of this study, and was consistent with the results from ecosystems with lower Ts and higher Q_10_ (Kirschbaum 1995; Blanke 1996). In addition, although the average Ts didn’t change significantly and the increasing trend was small, Rs of period 2 did not increase with Ts, but decreased sharply. The rate of rising Ts was relatively higher in winter than summer and the winter is the inactive period for Rs. Therefore, the rapid change of Rs in period 2 wasn’t caused by Ts.

**Figure 6.**
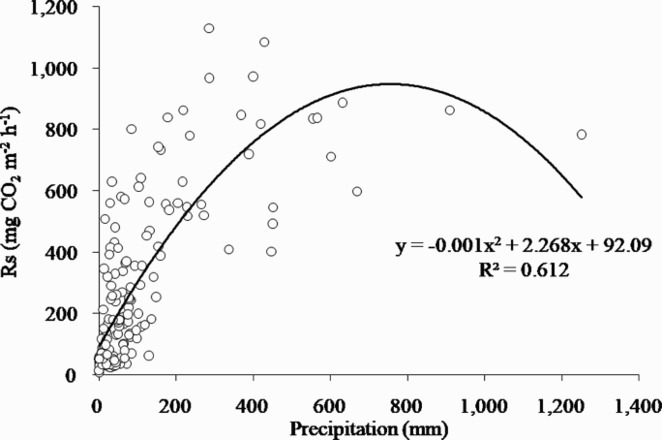
Correlation between monthly soil respiration (Rs) with monthly precipitation from 2004 to 2016 in a Korean cool-temperate deciduous forest. The relationship had a mostly positive correlation, but it was negative when precipitation exceeded the threshold.

In addition to the finding that the decline in the activity of soil microbes and roots by low precipitation reduced soil respiration (Pietikainen et al. 1999; Maier and Kress 2000), we confirmed that changes in precipitation and Rs occurred at the same time. The variation in precipitation was much larger than Ts. Thus, we determined that Rs was abruptly reduced. In addition, as shown in Figure 5, there was a difference between Rs values at similar Ts values. To analyze this result, Ts values were separated at 5°C intervals. The difference between the maximum and minimum Rs was 192–786 mgCO_2 _m^−2 ^h^−1^ each interval. As shown in the graph, the higher Ts value, the greater the difference. Thus, there was a difference in precipitation even in the same Ts range. In particular, in summer with high Ts values, when the difference in precipitation was larger because of the monsoon climate and there was a tendency for lower precipitation, the smaller Rs. It was concluded that the difference in precipitation affected Rs positively under a Ts constraint, and the lower precipitation, the smaller Rs. In addition, the relationship between monthly precipitation and Rs was a mostly positive correlation, but it was negative when precipitation exceeded the threshold (approximately 756 mm) (Figure 6). Therefore, it was concluded that the decrease in Rs in period 2 was caused by the decrease in precipitation.

Previous studies analyzed short-term and temporal data, and the researchers predicted that Ts was a major regulator of Rs (Hashimoto et al. 2009; Malcolm et al. 2009), and that the increase in Ts because of global warming will cause an increase in Rs (Schimel 1995; Rustad et al. 2000). However, in this study, we observed climate changes on the Korean Peninsula where the increase in temperatures in winter and a sudden change in the precipitation changed the soil moisture. Rs exhibited a remarkable change related to the change in precipitation. Thus, it is suggested that the results of previous studies and their predictions lack accuracy. Therefore, to predict the change in Rs resulting from climate change in the cool-temperate monsoon climate, long-term and continuous measurements should be conducted with simultaneous consideration of the effect of the precipitation, which have an ultimate effect on the soil moisture, in addition to temperature changes.

## 4. Conclusion

Soil respiration in a Korean cool-temperate deciduous forest, measured using the AOCC system from 2004 to 2016, showed a remarkable low value period from 2012 to 2016 compared with it from 2004 to 2011. During the same period, air and soil temperature increased gradually. The increase in temperature was greater in winter, which is the inactive period for soil respiration, than in summer, which is the active period. It was concluded that it did not affect the decrease in soil respiration However, precipitation decreased sharply with the change similar to the change in soil respiration after 2012, and was approximately 69% of the previous period from 2004 to 2001. In addition, unlike soil temperature, the change in precipitation appeared in summer, the active period of soil respiration. Therefore, it was concluded that the effect of precipitation on soil respiration after 2012 was higher than that of soil temperature. In previous studies, could not have detected these obvious changes in climate and soil respiration in the Korean peninsula observed in this study. This suggests that, to predict changes in soil respiration in cool-temperate monsoon climate, long-term and continuous measurement should be conducted considering the effect of precipitation along with that of temperature.
